# Development of Novel Oral Formulations of Disulfide Antioxidants Based on Porous Silica for Controlled Release of the Drugs

**DOI:** 10.3390/ma14040963

**Published:** 2021-02-18

**Authors:** Ekaterina S. Dolinina, Elena V. Parfenyuk

**Affiliations:** Laboratory “Chemistry of Hybrid Nanomaterials and Supramolecular Systems”, G.A. Krestov Institute of Solution Chemistry of Russian Academy of Sciences, 153045 Ivanovo, Russia; evp@isc-ras.ru

**Keywords:** silica, sol–gel synthesis pH, antioxidant-silica composites, surface modification, release kinetics and mechanism, GIT transit modeling, antioxidant activity

## Abstract

Powerful antioxidant α-lipoic acid (LA) exhibits limited therapeutic efficiency due to its pharmacokinetic properties. Therefore, the purpose of this work was to evaluate the ability of silica-based composites of LA as well as its amide (lipoamide, LM), as new oral drug formulations, to control their release and maintain their therapeutic concentration and antioxidant activity in the body over a long time. The composites synthesized at different sol–gel synthesis pH and based on silica matrixes with various surface chemistry were investigated. The release behavior of the composites in media mimicking pH of digestive fluids (pH 1.6, 6.8, and 7.4) was revealed. The effects of chemical structure of the antioxidants, synthesis pH, surface chemistry of the silica matrixes in the composites as well as the pH of release medium on kinetic parameters of the drug release and mechanisms of the process were discussed. The comparative analysis of the obtained data allowed the determination of the most promising composites. Using these composites, modeling of the release process of the antioxidants in accordance with transit conditions of the drugs in stomach, proximal, and distal parts of small intestine and colon was carried out. The composites exhibited the release close to the zero order kinetics and maintained the therapeutic concentration of the drugs and antioxidant effect in all parts of the intestine for up to 24 h. The obtained results showed that encapsulation of LA and LM in the silica matrixes is a promising way to improve their bioavailability and antioxidant activity.

## 1. Introduction

Oxidative stress is the basis of many pathological processes in the body, leading to various diseases and, ultimately, aging [1,2,3]. Antioxidants are molecules that can slow down or prevent oxidation in cells.

**Figure 1:**
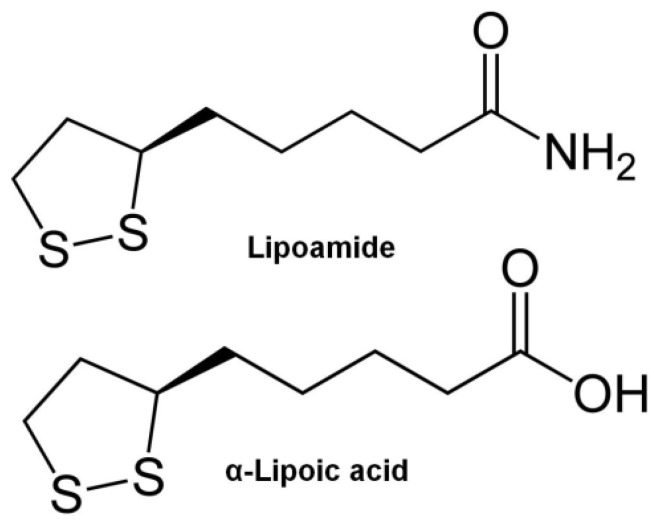
Structural formulas of antioxidants.

α-Lipoic acid (LA) and its amide (α-lipoamide, LM) (Figure 1) are powerful antioxidants. They are already used clinically or proposed for the treatment and prevention of various diseases [4,5,6,7,8,9,10,11].

However, LA is rapidly absorbed in the GIT, exhibits extensive presystemic metabolism, and has a low half-life in the body (0.5–1.5 h) [11,12,13]. The information about the properties of LM was not found in literature. The indicated pharmacokinetic properties lead to the need to take large doses of the drug several times a day. It is inconvenient for the patients taking the drugs orally. Daily intravenous drug injections are stressful, especially in children. In addition, long-term use of high doses of LA can lead to side effects [6,14,15]. Therefore, development of novel oral formulations of LA and LM with modified release is a particularly important problem. By providing for controlled release of the antioxidants from novel oral formulations, lowing the absorption and maintaining a desire level of the drugs and, hence, physiological effect over long period of time can be achieved.

Encapsulation (loading) of drugs in biologically relevant materials is promising way to create novel drug formulations with improved physicochemical and pharmacological properties. The encapsulation of LA in chitosan beads promoted sustained release of the drug in vitro [16,17]. The evaluation of LA–chitosan formulation in healthy volunteers showed that the sustained release was pulsed and an increased plasma level of a-lipoic acid can be achieved by this formulation for at least 12 h [18]. The results reported by Kofuji et al. demonstrated stabilization of LA against heat and light and controlled release of the drug encapsulated in alginic acid and chitosan beads [19]. The loading of LA in poly (lactic-co-glycolic acid) (PLGA) microspheres also led to controlled release of the drug and partially recovery of the mesenchymal stem cell proliferation and differentiation due to ROS scavenger activity of the antioxidant [20]. The works devoted to encapsulation of LA in lipid particles showed that the developed formulations might improve stability of the drug and elongate the half-life of the active substance [21,22]. The encapsulation of LA in surfactant micelles increased its thermal stability [23], and encapsulation of the drug in cyclodextrin enhanced its thermal stability and aqueous solubility [24].

Colloid silica is promising material for creation of new drug formulations. Especially, silica materials are suitable for the development of oral drug formulations, because colloid silica is biodegradable and recognized as safe food additive [25]. Furthermore, silica materials have high mechanical, thermal, photo stability, and resistance to the action of enzymes and microbial attacks. Despite these advantages over the materials mentioned above, no works have been found in the literature on the development of new formulations of the antioxidants based on colloid silica. It should be emphasized that there is no information in literature on development of any novel formulations of LM, although some studies indicate that LM is a more effective antioxidant than LA [5,8,26]. Taking into account the mentioned above facts, the purpose of this research was to develop new silica-based formulations of the antioxidants for oral administration, capable of releasing the drug in a controlled manner over a long time regardless of the pH of the release medium. In our previous works [27,28], a detailed description of the synthesis of silica-based composites of the antioxidants was given, and an extensive study of various properties of the composites and their silica matrixes (FTIR, UV spectra, zeta-potentials, XRD analysis, SEM, thermal properties, photostability) was performed. The drug–silica interactions were discussed. In this work, the release properties of LA and LM encapsulated in silica materials in release media with different pH were evaluated.

The composites were synthesized using sol–gel technology. It is well known that an important factor influencing the kinetics of the release of encapsulated drugs is their interaction with the capsule material. Literature data show that electrostatic interactions [29,30], hydrophobic interaction [31], hydrogen bonding [30,32], and π–π interaction [33] between drugs and silica matrix in silica-based formulations can promote sustained release of the drugs. Therefore, in this work, the effect of silica matrix modification on the functional properties of the composites was studied. Additionally, the example of the LA–silica composites was used to study the effect of the synthesis pH on the release behavior of the antioxidant.

The replacement of OH group in LA molecule with an amino group leads to changes in properties of LM compared to LA (acid–base properties, hydrophobicity, etc.), which, in turn, should have an effect on the physicochemical and functional properties of their composites with silica. Therefore, in this work, the effect of chemical structure of the antioxidants on the release properties was evaluated.

The release kinetics from the prepared composites was investigated using two methods. Firstly, the individual release profiles of LA and LM from the composites in buffer solutions with pH 1.6, 6.8, and 7.4 mimicking digestive fluids were obtained in order to determine mechanisms of the release processes and elucidate the effect of release medium pH on LA and LM release kinetics. On the basis of comparative analysis of the obtained results, the most promising composites were chosen. In order to mimic an in vivo situation, modeling of LA and LM release process from the composites with regard to the change in pH and residence time of the drugs in different segments of the gastrointestinal tract (GIT) (pH values corresponding stomach, segments of small intestine and colon, transit time) [34,35,36] was performed. Additionally, since the pharmacological effect of the drugs is determined by their antioxidant activity, the antioxidant effect of LA and LM was evaluated during the passage of the composites through the GIT. The antioxidant activity was investigated by using the DPPH method.

## 2. Materials and Methods

### 2.1. Reagents

DL-α-Lipoic acid (LA) (>99.0%) (CAS 1077-28-7) was purchased from Tokyo Chemical Industry Co., LTD (Tokyo, Japan). (±) α-Lipoamide (LPA) (96%) (CAS 940-69-2) was purchased from Toronto Research Chemicals (Toronto, ON, Canada). Tetraethoxysilane (TEOS) (ECOS, high purity grade, Moscow, Russia), ethanol (EtOH) (95%, Chimmed, analytical grade, Moscow, Russia), (3-mercaptopropyl)trimethoxysilan (MPTMOS) (95%) (CAS 4420-74-0), (Aldrich, St. Louis, MO, USA), (3-aminoporopyl)triethoxysilane (APTEOS) (99%) (CAS 919-30-2) (Aldrich, Kenilworth, NJ, USA), methyltrimetoxysilane (MTMOS) (98%+)(CAS 1185-55-3) (Acros Organics, New Jersey, NJ, USA), and 2,2-Diphenyl-1-picrylhydrazyl (DPPH) (CAS 1898-66-4) (Tokyo Chemical Industry Co., LTD, Tokyo, Japan) were used without further purification. Sodium dihydrogen phosphate (NaH_2_PO_4_·2H_2_O) and disodium hydrogen phosphate (Na_2_HPO_4_·12 H_2_O) (Chimmed, analytical grade, Moscow, Russia) were used to prepare buffer solutions with pH 6.0, 6.5, 6.8, and 7.4. Citric acid (Chimmed, analytical grade, Moscow, Russia), sodium hydroxide (Chimmed, analytical grade, Moscow, Russia), and hydrochloric acid (Acros, for analysis, 37%, Geel, Belgium) (CAS 7647-01-0) were used to prepare buffer solution with pH 1.6. The buffer solutions were prepared using doubly distilled deionized water.

### 2.2. Syntheses of LA and LM Composites with Different Silica Materials

The LA–silica or LM–silica composites were synthesized as described in [28]. In brief, solutions of LA or LM in ethanol (250 mg in 7.5 mL) were prepared by heating to 45 °C and ultrasonic treatment. The drug solution was introduced under stirring (600 rpm) in pre-synthesized silica sol obtained using TEOS or its mixture with a modifier (MPTOS (Aldrich, St. Louis, MO, USA), APTEOS (Aldrich, Kenilworth, NJ, USA), MTMOS (Acros Organics, New Jersey, NJ, USA)) and adjusted to pH 3 or 7 by using 2 M NaOH. The LM–silica composites were prepared at pH 7 only. Upon the synthesis of the composites with aminopropyl modified silica, pH of sol was adjusted to pH 3 or 7 by using 2 M HCl. After aging (seven days), the obtained composites were dried at room temperature for 2–3 days.

The designation of the synthesized composites and conditions of their synthesis are presented in Table 1.

As LA and LM are photosensitive, the synthesis, storage, and investigation of the drugs and their composites with the silica materials were carried out in the dark.

### 2.3. Particle Size Analysis

The average size and size distributions of the composite particles were determined by dynamic light scattering (DLS) with a Zetasizer Nano ZS (Malvern Instruments Ltd., Malvern, UK). Before DLS measurements, the samples were dispersed in deionized water and sonicated for 10 min to destruct agglomerates.

### 2.4. In Vitro LA and LM Release Measurements and Mathematical Analysis of Release Data

#### 2.4.1. Individual Release Profiles into the Media with pH 1.6, 6.8, and 7.4

The release profiles of the antioxidants were obtained by incubation of 0.25 g of the composite in 100 mL 50 mM buffer (pH 1.6, 6.8, 7.4) under stirring (100 rpm) at 37 °C. At appropriate time intervals, 5 mL samples were withdrawn and replaced by fresh buffer. The withdrawn samples were centrifuged at 10,000 rpm for 10 min. The amount of released drug was calculated from the absorbance at a wavelength of 332 nm measured spectrophotometrically (UV/VIS spectrometer Cary 100, Varian, Melbourne, Australia) using the calibration curves obtained at given pH. The release profiles were obtained by plotting the cumulative percentage of released drug as function of release time.

#### 2.4.2. Modeling the Release of LA and LM with Regard to the Change in pH and Residence Time of the Drugs in Different Segments of the GIT

Firstly, 0.25 g of the composite was suspended in 100 mL of aqueous HCl solution with pH corresponding to gastric juice pH (1.6 ± 0.2). The suspension was stirred (100 rpm) at 37 °C. Aliquots of 5 mL were withdrawn at fixed time intervals, centrifuged at 10,000 rpm for 10 min, and assayed by UV/VIS spectroscopic method to determine the amount of dissolved drug using calibration plots at given pH. The sample volumes were replaced with the same amounts of fresh medium. After release time corresponding to mean transit time through stomach (2 h) [34], the solution pH was adjusted to the mean pH in the proximal small intestine (6.0 ± 0.3) and then to the mean pH in distal part of the small intestine (7.4 ± 0.2) and colonic pH (6.5 ± 0.3) [33,34,35] by addition of several drops of 2M NaOH and/or 0.02 M NaOH. According to literature data, the transit time in small bowel in healthy humans is 3–6 h [34]. In the present study, the release times in the media simulating pH of proximal small intestine and its distal part were 3 and 3 h, respectively. The colon transit time is typically longer than the gastric and small bowel transit times and can amount up to 72 h [34]. In this work, the drug release in the medium simulating pH of colon was measured for 16 h. The amounts of released LA and LM in media simulating pH of the small intestine and colon were measured as described above.

#### 2.4.3. Analysis of Release Data

The release data were analyzed by model-dependent approach. Before the fitting the experimental release profiles to kinetic models, the burst release (M_b_) and time (t_b_) as well as maximum amount of the released drugs (M_∞_) were determined from the concentration dependences of the released antioxidants as has been described earlier [37]. They are presented in Table 2. The experimental release profiles after cutoff of the burst effects were fitted to the kinetic models. Four models, such as the zero order (Equation (1)) and the first order (Equation (2)) models, the Hixon–Crowell (Equation (3)), and the Korsmeyer–Peppas (Equation (4)) models [38] were applied to fit the experimental release profiles of the antioxidants:

(1) $$Q_{t}=Q_{0}+k_{0}\times t$$

(2) $$Q_{t}=Q_{0},\;e^{k_{1}\times t}$$

(3) $$Q_{0}^{1/3}-Q_{t}^{\frac{1}{3}}=k_{H\text{-}C}\times t$$

(4) $$\frac{M_{t}}{M_{\infty }}=k\times t^{n},\;\frac{M_{t}}{M_{\infty }}\leq 0.6$$

here *k*_0_, *k*_1_, *k_H-C_*, and *k*, are the zero order, the first order, the Hixon–Crowell, and the Korsmeyer–Peppas constants, respectively; *Q_o_* and *Q_t_* are the initial amount of drug in solution and the cumulative amount of drug released at time *t*, respectively; *M_t_*, *M_∞_* are the cumulative amount of released drug at time *t* and overall released amount, respectively; *n* is the exponent of release (related to the drug release mechanism) in function of time *t*.

The listed models are often used for description of drug release from porous matrixes and give very important information about kinetic law and parameters of drug release as well as mechanisms of the process.

### 2.5. In Vitro Antioxidant Activity Evaluation of LA and LM

In this work, the antioxidant activity of LA and its amide with regard to the change in pH and residence time of the drugs in different segments of the GIT was evaluated. Antioxidant activities of LA and LM in the release media were evaluated through a free radical-scavenging effect of stable DPPH. For this purpose, 0.01 g DPPH was dissolved in ethanol (200 mL), which was designed as pure DPPH solution. For determination of radical scavenging activity, pure DPPH solution was added to each sample in 1:1 ratio. The reaction mixture was incubated at room temperature for 15 min, and the absorbance at 515 nm was measured against blank samples. Decreased absorbance of the reaction mixture indicated increased superoxide anion scavenging activity. All data are an average of triplicate analyses. The antioxidant activity was calculated in percentage using the Equation (5):

(5) $$\mathrm{AA}\%=\left(1\;-\;\frac{A_{s}}{A_{0}}\right)\times 100$$

### 2.6. Statistics

All the experiments were performed in triplicate. The experimental results are expressed as the mean ± standard deviation (*n* = 3). For release data, linear or non-linear least-squares regressions were performed, and the model parameters were calculated. The model that best fit the release data was evaluated based on the correlation coefficient (R_2_). In some cases, the fitting experimental release profiles with the zero-order and the first-order models showed close values of correlation coefficients (R_2_). Therefore, the model that described the data properly was determined on the basis of comparison of the root mean square error (RMSE) (Equation (6)) [39] and Akaike Information Criterion (*AIC*) (Equation (7)) [40]. The criteria were calculated as

(6) $$RMSE=\sqrt{\frac{\sum_{i=1}^{n}{\left(y_{i}-y_{i\;\mathrm{mod}}\right)}^{2}}{n}}$$

(7) AIC=m lnSSR+2×p

where *y_i_* and *y_i_*_mod_ are the experimental and predicted by model ith values of variable, *m* is the number of data points, and *p* is the number of parameters of model. The model that shows the minimal values for the *SSR*, *RMSE*, and *AIC* gives the best description of the release data.

## 3. Results and Discussion

### 3.1. In Vitro Release Properties of Synthesized Composites in the Media with pH 1.6, 6.8, and 7.4

**Figure 2:**
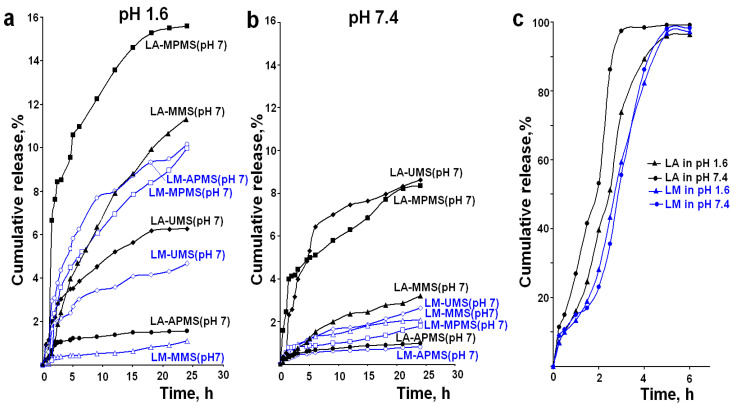
Release profiles of LA–silica composites (black line) and LM–silica composites (blue line) in the media with pH 1.6 (**a**) and 7.4 (**b**). Release profiles of LA and LM from pure powder samples in the media with pH 1.6 and 7.4 (**c**). The release data are presented as mean values of three measurements).

The purpose of this work was to evaluate the ability of silica-based composites of the drugs to control their release and maintain therapeutic concentration and antioxidant effect in the body over a long time. In order to develop such composites, it is necessary to select optimal silica matrix and optimize synthesis conditions of the composites. For this purpose, the composites of LA and LM with silica materials having various surface chemistry were prepared at different sol–gel synthesis pH, and their release properties were investigated. As an example, Figure 2 shows the experimental release profiles of LA and LM from the synthesized composites in the release media with pH 1.6 and 7.4. The release profiles show that the encapsulation of LA and LM in the silica materials facilitates sustained release of the drugs.

The elucidation of the effects of chemical structures of the drugs, surface chemistry of the silica matrixes, pH of sol–gel synthesis, as well as pH of release media on kinetics and mechanisms of the drug release was the main task of this work. However, the comparative analysis of the obtained data showed that, in most cases, it is impossible to elucidate the individual effects, because they overlap, and the observed phenomena are the result of influence of two or more effects on the drug release. Nevertheless, some interesting facts and regularities were revealed.

Table 2 shows the calculated values of burst effect, burst time, and maximum amount of the released drugs during 24 h. As can be seen from Table 2, the release of LA and LM is accompanied by a low burst effect (the maximum M_b_ is 3.9%). Usually, the burst effect is attributed to release of the drug located on or near the surface of particles and weakly bound to the matrix. The data presented in Table 2 showed that the maximum amounts of the released drugs for 24 h do not exceed 16% and 10%, respectively. This value for LA is significantly lower as compared to the acid encapsulated in chitosan microbeads and released in buffer solutions mimicking the GI tract for 8 h [16] or LA encapsulated in lipid nanoparticles and released in a phosphate buffer (pH 5.8) contained 20% ethanol for 12 h [22]. However, the result close to ours was observed by Lasoń et al. for release of LA encapsulated in another nanostructured lipid carriers in buffer solution (pH 7.4) for 24 h [21]. The authors of this work explained the sustained release by diffusion release mechanism of LA from these carriers. The sol–gel derived silica is non-swelling material and in general, more stable in the biological fluids than, for example, polysaccharides. In addition, other factors (synthesis conditions, drug loading, porous structure, etc.) can influence the mentioned parameter of release.

The APMS-based composites exhibited the lowest release of the drugs (not greater than 3.5%) in all tested release media, with the exception of the LA–APMS (pH 3) and LM–APMS (pH 7) composites in the medium with pH 1.6. (Table 2). These results can be explained by specific conditions of particle formation of the APMS-based composites, as well as the effect of strongly acidic medium on the release process.

**Figure 3:**
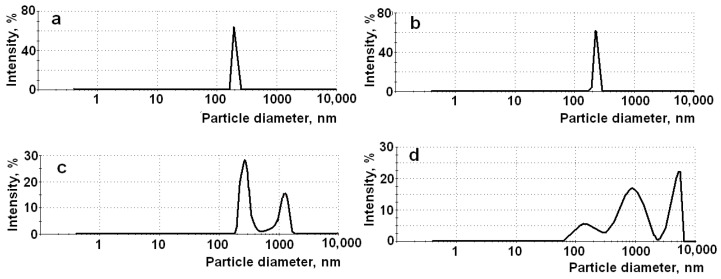
Particle size distribution for LA–UMS (pH 3) (**a**), LA–UMS (pH 7) (**b**), LA–APMS (pH 3) (**c**), and LA–APMS (pH 7) (**d**).

In contrast to other composites, upon synthesis of the APMS-based composites, solutions of the antioxidants were added in the reaction mixtures with high pH due to a very high basicity of 3-aminopropyl triethoxysilane (APTEOS) used as modifier of the silica matrix. Then, the pH of the resulting mixtures was quickly adjusted to final values (pH 3 or pH 7) by addition of HCl. The indicated conditions led to formation of the composites having higher particle size and polymodal particle size distributions (Table 3). Figure 3 demonstrates the particle size distributions for the LA–UMS (pH 3), LA–UMS (pH 7), LA–APMS (pH 3), and LA–APMS (pH 7) as example. As can be seen from Figure 3, the LA–UMS composites are nanosized and show narrow particle size distribution, whereas the LA–APMS (pH 3) exhibits the peak of nanosized particles and the peak of microparticles. The particles of the LA–APMS (pH 7) are mainly microsized. This fact is confirmed by the scanning electron microscopy study of the LA–silica composites reported earlier [28]. The higher particle size and consequently lower surface area of the composites led to the lower amounts of the released active substances.

In addition to the low amount of the released drugs, the indicated APMS-composites exhibited the lowest release rate. In order to determine the kinetic parameters and mechanisms of release of the antioxidants, the experimental release profiles were fitted with different kinetic models. The obtained results are presented in Table 4. The data presented in Table 4 show that the release profiles of the drug from the APMS-based composites exhibit the best fit with the first-order kinetic model. Due to higher contact surface between the particles of the large size, the APMS-based composites have higher condensed structures and, hence, higher stability in the release media. The matrixes do not degrade during the release process for 24 h (this is confirmed by a bad fit of the release profiles to the Hixon–Crowell model, which testifies about changes in the surface of the samples during the release process [38]), and the drugs release due to pseudo-Fickian diffusion mechanism (the Korsmeyer–Peppas diffusion exponent *n* < 0.43) [41,42]. The same effect of silica particle size on release of encapsulated drugs was reported in a number of works [43,44,45].

As for specific behavior of the LA–APMS (pH 3) and LM–APMS (pH 7) composites in the medium with pH 1.6, it can be attributed to weak hydrogen bonds between neutral forms of the drugs (pKa LA 4.7–5.1 [46]; LM is unionized at any pH [47,48]) and protonated APMS matrixes [28], which are easily destroyed in the highly acidic release medium.

The effect of sol–gel synthesis pH on the release kinetics and mechanisms was especially pronounced for the MMS-based composites. As can be seen from Table 4, the release from the LA–MMS (pH 3) composite (except the release into the medium with pH 6.8) is characterized by the *n* values about 0.24, indicating a pseudo-Fickian diffusion mechanism, and follows the first order kinetics. The pseudo-Fickian (retarded) diffusion can be a consequence of relatively strong interaction (hydrogen bonds) of uncharged LA with the weakly deprotonated matrix in the LA–MMS (pH 3) composite [28]. However, the release from the LA–MMS (pH 7) composite, as well as the LM–MMS (pH 7) composite (Table 4), is controlled by anomalous diffusion (*n* = 0.51–0.84) and shows a good fit to the zero order kinetic model. As applied to the composites based on porous non-swelling matrixes, the mechanism of anomalous diffusion includes the simultaneous action of two mechanisms: diffusion of a drug through the pores of the matrix to the surface of particles and disintegration(erosion)/degradation of the matrix [37,49,50,51]. The disintegration/degradation of the silica matrix is confirmed by a good fit of the release profiles of the MMS (pH 7)-based composites to the Hixon–Crowell model (R^2^ = 0.96–0.97) (Table 4). It is likely that the lower stability of the MMS (pH 7) matrix in the release media is attributed to a decrease in reactivity of the modifier, methyltrimetoxysilane (MTMOS), with increasing sol–gel synthesis pH [52,53]. Because the methyl group is electron-donating, the silanol (Si-OH) acidity is reduced in MTOS, and increasing sol–gel synthesis pH leads to a decrease in condensation rate, which results in formation of less condensed structures [52].

The effect of chemical structure of the studied antioxidants on their release from the composites is manifested in the fact that, in most cases, the release rate and the maximum amount of the released drug from the composites prepared under the same conditions are higher for LA than for LM, regardless of the pH of the release medium. This effect is demonstrated in Figure 2, which shows the release profiles of LA and LM in the media with pH 1.6 and 7.4, as example. The special behavior of LM composites should be noted. The release of LM from the most composites is higher in the medium with pH 1.6 in comparison with pH 7.4 (Figure 2). Furthermore, the release of LM in the media with pH 6.8 and 7.4 is very low and practically independent of the surface chemistry of the silica matrixes. The substitution of the OH in the carboxylic group of LA for an amino group leads to a significant decrease in acidic properties of LM as compared with LA due to conjugation of the lone pair of electrons of the nitrogen atom with π-electrons of C = O bond in the amide group. Furthermore, as an amide, LM exists as two resonance structures [47]. It is likely that these features of electronic structure of LM make it more stable and much less acidic than LA and lead to the lower solubility of LM in an aqueous medium compared to LA (solubility of LA is 0.127 mg/mL [46]; solubility of LM is <0.1 mg/mL [48]) and the maximum amount of the released LM as compared to LA. It is possible that the lower solubility of LM is the main reason for the indicated features of its release.

### 3.2. In Vitro Release Profiles and Antioxidant Activity in Accordance to the GIT Transit Conditions

The analysis of the individual release profiles of the antioxidants allowed us to identify the features of the drug release in the media with given pH values that mimic biological fluids. However, when taken orally, a drug passes through various parts of the gastrointestinal tract (GIT), which differ in the acidity of the medium and the time of transit through them. One of the conditions of maintaining constant concentration of drug during its passage through the GIT is the independence of the drug release rate from pH and time of transit through the different parts of the GIT. In this work, the modeling of the process of release of LA and LM from the synthesized composites was carried, taking into account the change in the medium pH and residence time of the drugs in different segments of the GIT.

Based on the analysis of the data presented in Table 4, the LA–MMS (pH 7) and LM–MPMS (pH 7) composites were selected as the most promising for the modeling because

- the drug release from the composites follows the zero order kinetics in all tested media, i.e., the kinetic law of the release is uniform in all digestive fluids; the zero order release is ideal behavior for drug formulation, which allows for a constant quantity of drug to be released over an extended period of time, resulting in uniform and sustained drug delivery;
- the drug release is controlled by anomalous diffusion; this means that the silica matrixes disintegrate/degrade during the release process, and this promotes their rapid elimination from the body;
- the release rates in the medium with pH 6.8 and 7.4 are close, i.e., it is supposed that the drug release in intestine does not depend on pH; a higher release rate in the medium with pH 1.6 allows reaching a certain concentration level in the intestinal media.

**Figure 4:**
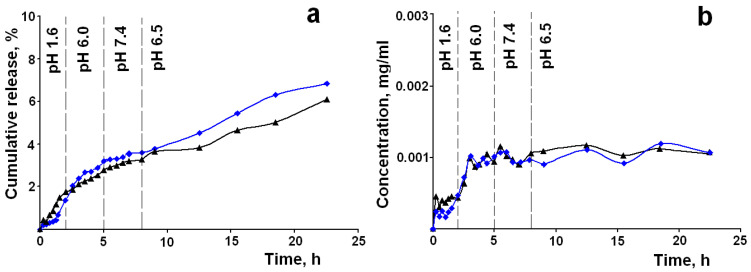
Model cumulative release profiles (**a**) and experimental concentration/time curves (**b**) of LA–MMS (pH 7) (black line) and LM–MPMS (pH 7) (blue line) composites upon changes in pH and residence time of the drugs in different segments of the GIT (results are average of triplicate experiments).

The cumulative release profiles and concentration/time curves for the LA–MMS (pH 7) and LM–MPMS (pH 7) composites mimicking the drug release according to the GIT transit conditions are presented in Figure 4.

The pH values in different segments of the GIT were chosen on the basis of analysis of literature data [32,33,34]. The model parameters of the release profiles are presented in Table 5.

The data in Table 5 show that, as expected, the release is close to the zero order kinetics and is controlled by anomalous (non-Fickian) diffusion regardless of pH and residence time of the drugs in different parts of the GIT. The concentration curves for LA and LM presented in Figure 4b are similar. After 3 h of release, the concentration of drugs in the medium of the small intestine reaches a value of 1.1 ± 0.2 μg/mL and is maintained at this level for up to 24 h, regardless of the pH of the medium and the transit time through different parts of the gastrointestinal tract. According to literature data, the concentration is close to the C_max_ in blood plasma after oral administration of different formulations of LA [54]. However, the C_max_ for the indicated formulations is reached after taking a dose of 600 mg, and the time to reach the maximum plasma concentration does not exceed 3 h. The samples of the studied composites contain only 61 mg and 46 mg per 1 g of the composites of LA and LM, respectively, but are able to maintain the indicated concentration in the release media for up to 24 h. Bernkop–Schnürch et al. [17] showed that the permeation of LA across the intestinal mucosa from guinea pigs was completely independent from the degree of the drug ionization, i.e., pH of the intestinal fluids. LM being an amide is mainly uncharged at any pH. Therefore, it may be assumed that the indicated factor will contribute to maintaining the therapeutic level of the released drugs in blood plasma.

**Figure 5:**
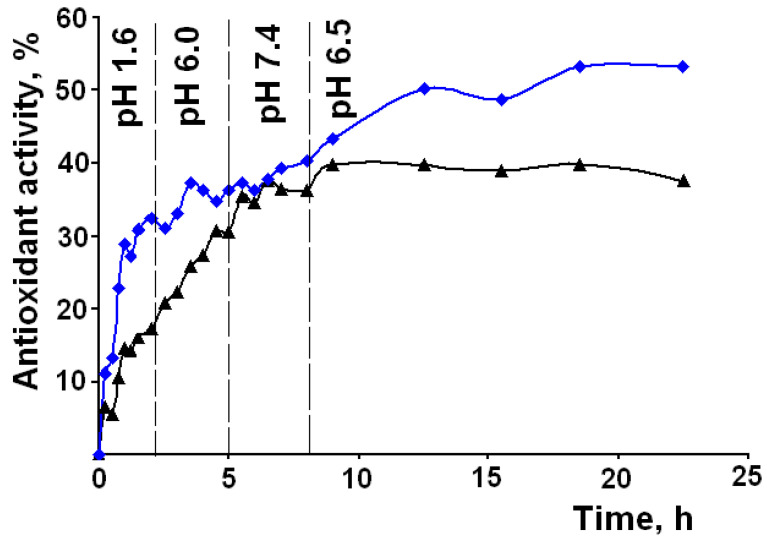
Experimental kinetic curves of antioxidant activity of the released LA (black line) and LM (blue line) from LA–MMS (pH 7) and LM–MPMS (pH 7) composites upon changes in pH and residence time of the drugs in different segments of the GIT (results are average of triplicate experiments.

It is well known that the therapeutic effect of LA and LM is associated with their antioxidant activity. It is interesting to see how the antioxidant activity of the released drugs will change with the changing pH of the biological fluids in different segments of the GIT. Figure 5 shows the experimental kinetic curves of antioxidant activity of the released LA and LM from the LA–MMS (pH 7) and LM–MPMS (pH 7) composites according to the GIT transit conditions. Some works reported that pH can influence antioxidant activity of drugs due to pH effect on their ionization [55,56,57]. When the pH changes, LA can ionize, because its pKa 4.7–5.1 [46]. However, ionization occurs at the carboxyl group in the side chain and does not affect the S–S bond in the 1,2-dithiolan ring, which is responsible for the antioxidant properties of the acid. LM is mainly unionized at any pH. Therefore, the antioxidant effect of LA and LM is mainly affected by their concentration in the release media. As can be seen from Figure 5, the changes in the antioxidant activity are similar to the changes in the concentration of the released drugs. The antioxidant effect is observed for a prolonged period of time and not just for a few hours. It should be noted that the antioxidant activity of LM is higher as compared to LA. After release for 24 h, the antioxidant effect of LM and LA are 53% and 38%, respectively.

## 4. Conclusions

Based on the comparative analysis of in vitro kinetics and mechanisms of the release of LA and LM from their composites with colloid silica with different surface chemistry and prepared at different sol–gel synthesis pH, it was shown that some composites can be a promising platform for development of novel oral formulations of the drugs with controlled release. The LA–MMS (pH 7) and LM–MPMS (pH 7) composites exhibited the release close to the zero order kinetics under conditions mimicking the drug transit through gastrointestinal tract. The composites containing only 61 mg and 46 mg/g of LA and LM, respectively, are able to maintain for up to 24 h a concentration similar to the concentration of LA in blood plasma after administration of 600 mg of some clinically used oral formulations. The antioxidant effect of the released drugs depends on the drug concentration in the release media. The composites exhibit the antioxidant effect for a long period of time (up to 24 h).

## Figures and Tables

**Table 1:** Designation of the synthesized composites and conditions of their synthesis.

Designation of Samples	Antioxidant	Precursor	Synthesis pH
LA–UMS (pH 3)	α-lipoic acid	TEOS	3
LA–UMS (pH 7)	α-lipoic acid	TEOS	7
LA–MPMS (pH 3)	α-lipoic acid	TEOS + MPTMOS	3
LA–MPMS (pH 7)	α-lipoic acid	TEOS + MPTMOS	7
LA–APMS (pH 3)	α-lipoic acid	TEOS + APTEOS	3
LA–APMS (pH 7)	α-lipoic acid	TEOS + APTEOS	7
LA–MMS (pH 3)	α-lipoic acid	TEOS + MTMOS	3
LA–MMS (pH 7)	α-lipoic acid	TEOS + MTMOS	7
LM–UMS (pH 7)	lipoamide	TEOS	7
LM–MPMS (pH 7)	lipoamide	TEOS + MPTMOS	7
LM–APMS (pH 7)	lipoamide	TEOS + APTEOS	7
LM–MMS (pH 7)	lipoamide	TEOS + MTMOS	7

**Table 2:** Drug loading, burst release (M_b_), burst time (t_b_), and maximum amount of the released drugs (M _∞_) in release media with pH 1.6, 6.8, and 7.4.

Composites	Drug Loading, mg/g ^a^	pH 1.6			pH 6.8			pH 7.4		
		M_b_, %	t_b_, h	M_∞_, %	M_b_, %	t_b_, h	M_∞_, %	M_b_, %	t_b_, h	M_∞_, %
LA–UMS (pH 3)	60	2.8	2.2	10.7	0.5	0.5	2.0	0.2	0.5	5.0
LA–UMS (pH 7)	57	1.2	1.1	6.2	1.7	0.9	4.1	0.6	1.2	8.6
LA–MMS (pH 3)	60	3.2	2.6	4.0	0.9	0.4	11.3	0.3	0.5	9.0
LA–MMS (pH 7)	61	1.3	1.4	10.5	0.4	0.5	2.5	0.1	0.4	3.2
LA–MPMS (pH 3)	59	0.2	0.5	4.4	0.5	0.5	12.1	0.6	0.5	3.8
LA–MPMS (pH 7)	58	0.6	0.9	15.6	1.0	1.5	2.4	3.9	1.5	8.4
LA–APMS (pH 3)	46	2.9	1.0	7.8	0.5	0.5	1.4	0.7	0.9	3.4
LA–APMS (pH 7)	56	0.7	1.4	1.6	0.2	1.5	0.5	0.3	1.1	1.0
LM–UMS (pH 7)	57	2.0	2.5	4.7	0.6	1.8	2.6	0.6	2.5	1.4
LM–MMS (pH 7)	44	0.1	1.1	1.1	0.4	1.5	2.1	1.0	2.1	1.8
LM–MPMS (pH 7)	46	0.3	1.5	9.8	0.8	1.9	1.8	0.6	2.0	1.3
LM–APMS (pH 7)	53	0.9	1.0	10.0	0.3	1.5	0.8	0.4	1.0	1.1

^a^ mg/g of composite; the uncertainty is 1%.

**Table 3:** Mean particle size of the synthesized composite.

Composite	Mean Diameter, nm	Composite	Mean Diameter, nm
LA–UMS (pH 3)		LA–APMS (pH 3)	
	201 ± 18;		189 ± 39; 1050 ± 98;
	PDI 0.072		PDI 0.639
LA–UMS (pH 7)		LA–APMS (pH 7)	
	201 ± 18;		173 ± 61; 911 ± 291; 4628 ± 644;
	PDI 0.072		PDI 0.897
LA–MMS (pH 3)	190 ± 15; PDI 0.043	LM–UMS (pH 7)	264 ± 19; PDI 0.076
LA–MMS (pH 7)	218 ± 21; PDI 0.074	LM–MMS (pH 7)	202 ± 17; PDI 0.053
LA–MPMS (pH 3)	198 ± 15; PDI 0.039	LM–MPMS (pH 7)	222 ± 22: PDI 0.102
LA–MPMS (pH 7)	222 ± 19; PDI 0.051	LM–APMS (pH 7)	
			165 ± 31; 863 ± 306; 6865 ± 716;
			PDI 0.788

**Table 4:** Model parameters of LA and LM release from LA–silica and LM–silica composites in different release media (The estimated uncertainty in *n* is 6%).

	pH 1.6				pH 6.8				pH 7.4			
	Zero-order model	First-order model	Hixon–Crowell model	Korsmey er–Peppas model	Zero-order model	First-order model	Hixon–Crowell model	Korsmey- er–Peppas model	Zero-order mode	First-order model	Hixon– Crowell model	Korsmey- er–Peppas model
**LA–UMS** **(pH3)** R^2^ *RMSE* *AIC*	*k*_0_ = 0.301 0.9509 5.38 65.44	*k*_1_ = 0.0014 0.9351 1.35 35.04	0.9338	*k* = 2.13 *n* = 0.44 0.9944	*k*_0_ = 0.047 0.9525 0.18 −9.74	*k*_1_ = 0.0002 0.9330 0.74 32.13	0.9588	*k* = 2.28 *n* = 0.47 0.9919	*k*_0_ = 0.151 9417 0.57 23.11	*k*_1_ = 0.0007 0.9439 2.26 61.73	0.9557	*k* = 1.91 *n* = 0.49 0.9872
**LA–UMS** **(pH 7)** R^2^ *RMSE* *AIC*	*k*_0_ = 0.192 0.9480 2.471 58.86	*k*_1_ = 0.0009 0.9465 0.789 27.16	0.9309	*k* = 2.04 *n* = 0.43 0.9850	*k*_0_ = 0.052 0.9211 0.634 23.48	*k*_1_ = 0.0002 0.9618 0.213 −4.84	0.8987	*k* = 1.72 *n* = 0.21 0.9779	*k*_0_ = 0.270 0.9303 3.838 70.32	*k*_1_ = 0.0012 0.9543 1.121 38.31	0.9245	*k* = 1.92 *n* = 0.44 0.9717
**LA–MMS** **(pH 3)** R^2^ *RMSE* *AIC*	*k*_0_ = 0.002 0.9245 0.399 6.69	*k*_1_ = 0.0001 0.9500 0.101 −20.75	0.9034	*k* = 1.85 *n* = 0.25 0.9548	*k*_0_ = 0.470 0.9930 1.916 5.17	*k*_1_ = 0.0022 0.9201 8.151 97.69	0.9889	*k* = 4.13 *n* = 0.98 0.9884	*k*_0_ = 0.198 0.9254 2.611 65.82	*k*_1_ = 0.0009 0.9567 0.771 31.66	0.9054	*k* = 1.38 *n* = 0.22
**LA–MMS** **(pH 7)** R^2^ *RMSE* *AIC*	*k*_0_ = 0.417 0.9655 1.805 45.98	*k*_1_ = 0.0019 0.9236 6.516 76.89	0.9755	*k* = 2.43 *n* = 0.71 0.9677	***k*_0_= 0.095** 0.9855 0.336 8.42	*k*_1_ = 0.0004 0.9845 1,226 44.68	0.9853	*k* = 3.29 ***n *= 0.77** 0.9879	***k*_0_ = 0.101** 0.9667 0.486 18.76	*k*_1_ = 0.0006 0.9409 1.886 56.81	0.9779	*k* = 2.81 ***n* = 0.78** 0.9739
**LA–MPMS** **(pH 3)** R^2^ *RMSE* *AIC*	*k*_0_ = 0.155 0.9452 0.579 26.24	*k*_1_ = 0.0014 0.9488 2.384 68.68	0.9663	*k* = 1.77 *n* = 0.46 0.9709	*k*_0_ = 0.285 0.935 4.751 89.37	*k*_1_ = 0.0008 0.9499 1.070 44.66	0.9004	*k* = 1.43 *n* = 0.25 0.9949	*k*_0_ = 0.135 0.9545 1.017 43.15	*k*_1_ = 0.0006 0.9377 3.719 88,75	0.9677	*k* = 2.92 *n* = 0.77 0.9837
**LA–MPMS** **(pH 7)** R^2^ *RMSE* *AIC*	*k*_0_ = 0.392 0.9063 6.129 89.81	*k*_1_ = 0.0019 0.9502 1.581 51.78	0.9088	*k* = 1.42 *n* = 0.25 0.9850	*k*_0_ = 0.059 0.9776 0.555 20.07	*k*_1_ = 0.0003 0.9636 3.420 67.31	0.9734	*k* = 3.59 *n* = 0.82 0.9749	*k*_0_ = 0.202 0.9887 0.806 29.74	*k*_1_ = 0.0009 0.9806 3.420 67.88	0.9891	*k* = 4.53 *n* = 0.95 0.9855
**LA–APMS** **(pH 3)** R^2^ *RMSE* *AIC*	*k*_0_ = 0.121 0.9099 2.022 53.64	*k*_1_ = 0.0006 0.9608 0.501 17.36	0.9007	*k* = 1.74 *n* = 0.25 0.9843	*k*_0_ = 0.020 0.9099 0.329 7.815	*k*_1_ = 0.0001 0.9536 0.080 −31.90	0.9034	*k* = 1.82 *n* = 0.23 0.9749	*k*_0_ = 0.116 0.9377 1.696 49.08	*k*_1_ = 0.0003 0.9666 0.472 15.95	0.9111	*k* = 1.33 *n* = 0.39 0.9855
**LA–APMS** **(pH 7)** R^2^ *RMSE* *AIC*	*k*_0_ = 0.028 0.9079 0.352 6.79	*k*_1_ = 0.0002 0.9577 0.116 −19.83	0.9101	*k* = 1.98 *n* = 0.33 0.9803	*k*_0_ = 0.011 0.9558 0.153 −13.13	*k*_1_ = 0.0001 0.9565 0.048 −41.37	0.9259	*k* = 2.10 *n* = 0.39 0.9588	*k*_0_ = 0.026 0.9556 0.326 6.23	*k*_1_ = 0.0001 0.9572 0.105 −23.36	0.9321	*k* = 2.16 *n* = 0.41 0.9755
**LM–UMS** **(pH 7)** R2 *RMSE* *AIC*	*k*_0_ = 0.113 0.9677 0.524 12.12	*k*_1_ = 0.0005 0.9377 2.256 41.30	0.9801	*k* = 3.28 *n* = 0.75 0.9675	*k*_0_ = 0.079 0.9700 0.341 4.68	*k*_1_ = 0.0003 0.9406 1.387 35.58	0.9704	*k* = 2.67 *n* = 0.64 0.9865	*k*_0_ = 0.037 0.9927 0.169 −10.47	*k*_1_ = 0.0002 0.9472 0.407 7.07	0.9921	*k* = 4.51 *n* = 0.92 0.9798
**LM–MMS** **(pH 7)** R2 *RMSE* *AIC*	*k*_0_ = 0.307 0.9714 0.137 −16.42	*k*_1_ = 0.0001 0.9344 0.480 10.18	0.9663	*k* = 3.72 *n* = 0.76 0.9565	*k*_0_ = 0.061 0.9600 0.262 −0.303	*k*_1_ = 0.0003 0.9433 0.797 26.38	0.9633	*k* = 2.13 *n* = 0.51 0.9677	*k*_0_ = 0.037 0.9766 0.162 −11.70	*k*_1_ = 0.0002 0.9703 0.495 12.92	0.9766	*k* = 4.32 *n* = 0.84 0.9577
**LM–MPMS** **(pH 7)** R2 *RMSE* *AIC*	*k*_0_ = 0.307 0.9688 1.330 38.67	*k*_1_ = 0.0014 0.9207 5.272 71.71	0.9764	*k* = 3.88 *n* = 0.79 0.9644	***k*_0_= 0.041** 0.9676 0.202 −6.86	*k*_1_ = 0.0002 0.9601 0.939 26.53	0.9773	*k* = 5.50 ***n*= 0.98** 0.9607	***k*_0_= 0.035** 0.9892 0.116 −17.94	*k*_1_ = 0.0001 0. 9555 0.408 8.66	0.9893	*k* = 2.95 ***n*= 0.67** 0.9612
**LM–APMS** **(pH 7)** R2 *RMSE* *AIC*	*k*_0_ = 0.309 0.8984 3.968 71.18	*k*_1_ = 0.0014 0.9716 1.297 42.11	0.9022	*k* = 1.84 *n* = 0.42 0.9825	*k*_0_ = 0.019 0.9104 0.247 1.73	*k*_1_ = 0.0001 0.9660 0.084 −27.53	0.9107	*k* = 2.26 *n* = 0.41 0.9650	*k*_0_ = 0.025 0.9126 0.377 10.03	*k*_1_ = 0.0001 0.9677 0.105 −23.23	0.9124	*k* = 1.80 *n* = 0.30 0.9752

**Table 5:** Model parameters of LA and LM release from LA–MMS (pH 7) and LM–MPMS (pH 7) composites taking into account the conditions of transit through the GIT.

Composite	Mb, %	Zero Order	First Order	Hixon-Crowell	Korsmeyer-
	tb, h	Model	Model	Model	Peppas Model
LA–MMS (pH 7)	1.5 1.4	*k*_0_ = 0.201 R^2^ = 0.9737	*k*_1_ = 0.0011 R^2^ = 0.9194	R^2^ = 0.9763	*k* = 1.93
					*n* = 0.64
					R^2^ = 0.9577
LM–MPMS (pH 7)	0.5 1.3	*k*_0_ = 0.244 R^2^ = 0.9577	*k*_1_ = 0.0012 R^2^ = 0.9455	R^2^ = 0.9661	*k* = 2.28
					*n* = 0.66
					R^2^ = 0.9585

## Data Availability

Data sharing is not applicable to this article.
